# Multisite musculoskeletal pain among young technical school students entering working life

**DOI:** 10.1186/s12891-016-0938-6

**Published:** 2016-02-16

**Authors:** Therese Nordberg Hanvold, Lars-Kristian Lunde, Markus Koch, Morten Wærsted, Kaj Bo Veiersted

**Affiliations:** National Institute of Occupational Health, P.O. Box 8149 Dep, 0336 Oslo, Norway

**Keywords:** Musculoskeletal disorders, Multisite pain, Young workers, Adolescents, Work-related factors, Longitudinal

## Abstract

**Background:**

There is a need to investigate the occurrence of multisite pain in young adults and to determine potential factors contributing to the early course of multisite musculoskeletal pain. The aim of this prospective study was to assess the occurrence and change of prevalence in the number of pain sites. We also wanted to identify work-related and individual risk factors associated with the number of musculoskeletal pain sites.

**Methods:**

We monitored musculoskeletal pain from 4 body regions, individual and work-related factors on 21 occasions over a 6.5 year period. The cohort consisted of 420 technical school students entering working life. Data were analyzed by generalized estimating equations (GEE).

**Results:**

Pain from more than one body site was prevalent in this cohort of young adults (69 % at baseline), and the number of body sites in pain was found quite stable over the 6.5 year follow-up period. Women had higher number of pain sites compared with men and gender specific risk factors were identified. Increased mechanical workload and quantitative demands and low socioeconomic status were associated with increased number of musculoskeletal pain sites among women, while tobacco use was found as a risk factor among young men. Increased perceived muscle tension was the only factor significantly associated with increased number of pain sites in both genders.

**Conclusion:**

The current study supports earlier findings and show that pain from multiple body sites are frequent also among young workers. The identification of gender specific risk factors in our study is important and may facilitate practical prevention and future research.

## Background

The majority of the research on work-related risk factors and musculoskeletal focuses on pain from specific anatomical sites. Mechanical workload such as awkward postures, manual material handling prolonged standing and awkward lifting are acknowledged as risk factors for localized musculoskeletal pain [1–3]. The research is based on an assumption that prolonged local mechanical load generates a local effect that leads to pain symptoms. At the same time there is increasing evidence that psychosocial factors such as high levels of job demands at work also play an important role for localized musculoskeletal pain [3, 4]. There is also growing attention on the fact that pain from only one specific anatomical site is found to be relatively rare and that pain from multiple body sites are more frequent [5–7]. Functional problems have been found to increase with increasing number of pain sites [8].

In working populations multisite pain has been found to be more severely related with work disability [6, 9] and long term sickness absence [10]. A recent study comprising of workers from 18 different countries found that when comparing workers who had pain at one anatomical site with workers who did not, the workers with pain were twice as likely to have pain in other sites [11]. The results also suggests that multisite pain differs in its associations with risk factors from pain limited to a single site [11]. Multisite pain have been found to have stronger associations with gender, age, somatising tendency and exposure to physically heavy work compared with single sited pain [11, 12]. A 14 year prospective study found that the number of pain sites appears relatively stable across adulthood and recommended investigating the occurrence of multisite pain in adolescents and young adults to determine potential factors contributing to the early course and development of multisite musculoskeletal pain [13].

The knowledge on occurrence and changes of multisite pain among young adults are sparse. So is the knowledge on work-related risk factors for multisite pain among young workers. The aim of this prospective study was therefore to assess the occurrence and change of prevalence in the number of pain sites over a 6.5 year period among young adults. In addition we wanted to evaluated gender differences and identify work-related and individual risk factors associated with the number of musculoskeletal pain sites among a cohort of technical school students entering working life.

## Methods

### Study population

In this 6.5 year prospective cohort study technical school students were followed from school (T0-T2), through their apprenticeship (T3-T9) and into working life (T10-T20) [14]. Four hundred and ninety six participants were invited at baseline and 420 participated, giving a response rate of 85 % at baseline. The 420 participants (153 men, 267 women, mean age 17.5 (±1.2) years), were recruited in their second year of technical school. Twenty-eight percent were student electricians, 40 % were student hairdressers and 32 % studied media and design. The study was approved by The Norwegian Data Inspectorate and the scientific ethical committee system. The participants were informed of the procedures and gave their written informed consent. For participants younger than 18 years at baseline, parental consent was also obtained.

### Data collection

The study was initiated in 2002 recruiting students from 13 different technical schools in the greater Oslo area in Norway. The data collection was conducted from October 2002 to February 2009. The baseline assessment (T0) comprised of a questionnaire and a clinical examination and took place at school during school hours. The follow-up questionnaires were sent to the participants approximately every 4th months, giving a total of 21 time points (T0-T20). Only the outcome measure; musculoskeletal pain was assessed at all the 21 time points. The variables considered as time-constant were only assessed once (socioeconomic status (T0), hand grip strength (T0), shoulder endurance (T0) and perceived muscle tension (T8)). The variables considered as time-varying were assessed at multiple time points (mechanical workload (T0,T1, T4, T7, T10, T11, T13, T14, T17, T20), psychosocial work factors (T7, T11, T14, T17, T20), tobacco use (T0,T7, T11, T14, T17, T20) and physical activity (T1, T2, T4, T5, T7, T11, T14, T17, T20)). All data were assessed by self-reported questionnaire, except the clinical assessment of hand grip strength and shoulder endurance.

### Measurements

#### Multisite musculoskeletal pain

The participant’s musculoskeletal pain for the preceding 4 weeks was assessed for 4 anatomical sites: neck-shoulder, low back, arm-hand-wrist and hip-knee-leg. The anatomical site was illustrated by a mannequin drawing [15]. For each anatomical site, pain intensity (no pain (0), mild pain (1), moderate pain (2) and severe pain (3)) were assessed [16]. The pain variable was thereafter dichotomized in no pain (0) or any pain (1–3). The number of body sites in pain was then computed by adding the number of painful anatomical sites from the 4 anatomical regions (0–4) [12].

#### Socioeconomic status

The participant’s socioeconomic status was assessed by one question “How wealthy do you consider your family?” The question had five response categories (0) very wealthy (1) wealthy, (2) average wealthy, (3) not particularly wealthy and (4) not wealthy [17]. To create comparable groups we dichotomized socioeconomic status in a low and high group. Low being those reporting lower than average wealth (3–4) and high being those reporting average wealth or better (0–2).

#### Tobacco use

The participants were asked about their smoking and snuff habits. If they either were smokers or used snuff daily or occasionally they were characterized as tobacco users.

#### Perceived muscle tension

The participants self-reported muscle tension was evaluated by 11 questions on muscle tension habits. The questions concerned whether the subjects had the habit of raising their shoulders, contracting their neck muscles, holding tools unnecessarily tensely, contracting their stomach muscles, wrinkling the forehead, contracting the eyelids, contracting the chewing muscles, holding their breath, shallow or strained breathing, sitting on the edge of the chair and grinding their teeth. Each question had 3 response alternatives ranging from 0 (never) to 2 (often) giving a muscle tension index ranging from 0–22 [18, 19].

#### Hand grip strength

Hand grip strength test was performed in standing position with the hands pointing downward. Each participant performed three maximal contractions with their dominant hand using a hand dynamometer, model 78010 from Lafayette Instrument® (Lafayette, IN 47903 USA). The highest of three attempts was recorded [20].

#### Shoulder muscle endurance capacity

The isometric endurance capacity in the shoulder muscles was quantified by the time (seconds) the participants could keep both shoulders abducted at 45° with a load of 2 kg on each wrist. They were asked to hold the position as long as possible. This was done in concordance with protocol from a previous study [21]. An upper limit was set at 900 s (15 min). Four participants reached this limit.

#### Physical activity level in leisure time

The level of physical activity in leisure time was measured by 1 question. The participants were asked how often they performed activities that led to increased heart rate and shortness of breath. The question had seven response categories ranging from 0 (never) to 6 (everyday) [17].

#### Work-related mechanical load

Twelve questions were used to assess the work-related mechanical exposure [22]. The participants were asked whether their work involved or required repetitive movements, precision movements, body postures such as working with their arms elevated or their back twisted. All 12 questions had three response alternatives; 0 (nothing/hardly nothing), 1 (somewhat) and 2 (a great deal) giving index ranging from 0–24. The index show good reliability (weighted kappa = 0.92, Cronbach alpha = 0.85) [22].

#### Work-related psychosocial factors

Quantitative demands and control over work intensity were each assessed by 2 questions. Items were selected from the General Nordic Questionnaire for Psychological and Social Factors at Work (QPS_Nordic_) [23]. The questions on quantitative work demand were “Is your workload irregular so that the work piles up?” and “Do you have too much to do?”. The questions assessing control over work intensity were “Can you set your own work pace?” and “Can you determine the length of your own breaks?”. All the questions had 5 response alternatives ranging from 0 (never/seldom) to 4 (often/very often). The mean of the 2 questions made the score for demand and control, respectively.

### Statistics

#### Data procedures

As this was a prospective study with measurements at 21 time points, missing data had to be analyzed and handled prior to statistical analyses. The majority of missing data constituted of intermittent missing as the participants could have missing data from one questionnaire in between complete questionnaires. The follow-up response rate ranged from 70 % (*N* = 292) at T2 to 27 % (*N* = 112) at T18. A total of 183 participants (44 %) answered more than half of the questionnaires in the follow-up period. The total loss to follow up was 7 % as 30 participants only answered the baseline questionnaire while the complete follow-up rate were 5 % as 21 participants answered all the 21 questionnaires. There was also missing data as some participants did not fill in single items in a questionnaire (item non-responders). Missing because of item non-responders ranged from 1 % at T20 to 9 % at T10 for mechanical workload. For musculoskeletal pain the item missing, ranged from 0.6 % at T20 to 2 % at T2. Multiple imputations of the missing data were done as we considered the missing as: missing at random. This assumption was based on an analysis were the baseline pain and work exposure showed no significant difference between those answering the last follow-up after 6.5 years (*N* = 193) and those who did not (*n* = 227). An attrition analyses was also done taking into account the reports during the follow-up period and the intermittent missing, and it showed no significant association between the amount of missing and musculoskeletal pain reports or the work exposure variables [24]. More detailed information on the missing data has been described elsewhere [14]. The imputation procedure consisted of multiple imputations of all missing values were done based on a linear mixed model [25]. The imputation model included all the variables which are used in the multivariate analysis in this study. Five imputed datasets were made and on the basis of them one average estimate was calculated [26].

#### Statistical analyses

Statistical analyses were done using STATA (version 12.0). To evaluate the change of prevalence in the number of pain sites and evaluate longitudinal associations we used Generalized Estimating Equations (GEE-analysis) taking into account the dependency of the observations within the individual by adding a “within-subject correlation structure”. Due to over dispersion in the outcome: number of pain sites, a negative binomial GEE-analysis was used. For the effect estimates, rate ratio (RR), with corresponding 95 % confidence intervals reported. In all negative binomial GEE-analysis an exchangeable correlations structure was used. The multivariate analyses were adjusted for covariates selected for inclusion a priori. A gender difference in the number of musculoskeletal sites in pain, led to analyses done both for the whole group and stratified by gender. In a supplementary analysis Cramer’s V (φ_c_) was performed to evaluate the association between the four musculoskeletal pain sites.

## Results

Pain reported from the four anatomical regions coexisted and showed statistically significant associations. The strongest associations were found between low back pain and hip, knee and leg pain (φ_c_: 0.22 *p* < 0.01) and between neck/shoulder pain and low back pain (φ_c_: 0.19 *p* < 0.01). Only 12 % of the 420 participants (*n* = 51) reported to be pain free (no pain in any of the four body sites) at baseline. Neck/shoulder and low back were the two body regions with highest prevalence of pain at baseline (Table 1). Assessing men and women separately showed that male participants more often than females reported to be pain free or having localized pain, while female participants reported higher prevalence of multisite pain compared to men.

**Table 1:** Prevalence of pain from 4 different body regions and number of body sites in pain (0–4), from baseline T0 (*N* = 420), by gender

	All (*n* = 420) N (%)	Men (*n* = 153) N (%)	Women (*n* = 267) N (%)
Pain sites			
Neck and shoulder	297 (71)	80 (52)	217 (81)
Low back	223 (53)	69 (45)	154 (58)
Arm, hand and wrist	135 (32)	46 (30)	89 (33)
Hip, knee and leg	204 (49)	60 (39)	144 (54)
Number of body sites in pain			
0	51 (12)	30 (19)	21 (8)
1	82 (19)	38 (25)	44 (17)
2	134 (32)	50 (33)	84 (31)
3	103 (25)	23 (15)	80 (30)
4	50 (12)	12 (8)	38 (14)

### Changes in the number of pain sites over time

**Fig. 1:**
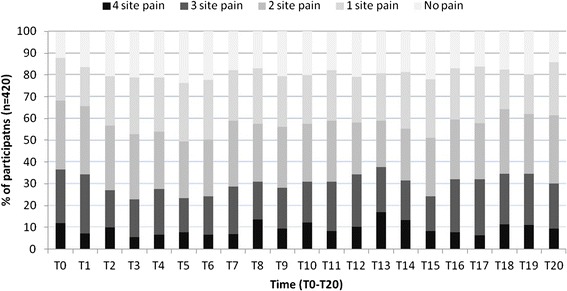
Prevalence of number of musculoskeletal pain sites among young adults in technical school (T0-T20). The bodily pain reports are categorized in five groups No pain, pain reported from one site, pain reported from 2 sites, pain reported from 3 sites and pain reported in all 4 site

During the whole study period pain from more than one anatomical body region was most commonly. In all 21 time points, except one (T5), more than 50 % reported 2, 3 or 4 body sites in pain (Fig. 1). The prevalence of musculoskeletal pain sites among young adults over the 6.5 year period can be assessed as quite stable on a group level. The prevalence of musculoskeletal pain sites among young adults over the 6.5-year period can be assessed as quite stable on a group level. The prevalence of reporting no pain changed from 12 % at baseline, 20 % at T10 to 14 % after 6.5 years (T20). Localized pain (only pain at one site) was reported by 19 % at baseline, 22 % at T10 and 25 % at T20. Pain from ≥2 body sites was reported by 69 % at baseline, 58 % at T10 and 61 % at T20. On an individual level, the consistency of the pain was also quite stable. Forty-seven percent reported pain from ≥2 body sites both at baseline and after 6.5 years, 7 % had localized pain at both occasions while 3 % had no pain at both baseline and the last follow-up. Changes in the number of body regions in pain was also found, 23 % changed between reporting multisite pain and localized pain while 13 % changed between multisite pain and no pain, 7 % changed between localized and no pain. The adjusted GEE analysis showed a slight increase in the number of body regions in pain over the study period on a group level (RR = 1.01 95 % CI 0.99–1.01, *P* = 0.05). Analyses stratified by gender show that this increase was only seen among the male participants as no significant effect of time was seen among women (Table 3). Changes in the time-dependent work-related factors were analyzed and no statistically significant changes in self-reported mechanical workload was found over the 6.5 year period (RR = 0.99, 95 % CI 0.99–1.00, *P* = 0.54). For the psychosocial work factors it was found a small but significant increase in both quantitative work demands (RR = 1.01, 95 % CI 1.00–1.01, *P* < 0.01) and control over work intensity (RR = 1.01, 95 % CI 1.00–1.01, *P* < 0.01) over the study period.

### Factors associated with the number of pain sites

#### All subjects

The analyses show that a high socioeconomic status have a protective effect on the number of pain sites reported (see Tables 2 and 3). Tobacco use, female gender, exposure to mechanical workload and an increased perceived muscle tension constituted a risk for reporting musculoskeletal pain from multiple sites. Women had 20 % increased number of musculoskeletal pain sites compared to men. Tobacco users reported a 16 % increase in number of pain sites than participants not smoking or using snuff. Each additional increase in mechanical workload (0–24) was associated with a 1 % increase in the number of pain sites reported. With regards to perceived muscle tension each additional increase in the tension score (0–22) was associated with a 2 % increase in the number of pain sites reported.

**Table 2:** The unadjusted GEE analyses of the association between musculoskeletal pain (0–4) and work-related and individual risk factors

		All (*N* = 420)		
Number pain sites		No. observations = 8820 (T0-20, 6½ years)		
		RR	95 % CI	*P*-value
Gender	Men	1.00		
	Women	1.26	1.15–1.39	<0.01
SO	Low	1.00		
	High	0.90	0.81–1.01	0.06
Tobacco use	No	1.00		
	Yes	1.16	1.06–1.26	<0.01
Mechanical workload (0–24)		1.00	1.00–1.01	0.01
Control over work intensity (0–4)		1.01	0.99–1.02	0.46
Quantitative work demands (0–4)		1.01	0.99–1.03	0.17
Perceived muscle tension (0–22)		1.03	1.02–1.04	<0.01
Handgrip strength (kg)		0.99	0.98–0.99	<0.01
Muscle endurance (Seconds)		0.99	0.99–0.99	0.02
Physical activity (0–6)		0.99	0.98–1.00	0.07
Time (T0-T20)		1.00	0.99–1.00	0.06

[RR = rate ratio, CI = 95 % confidence interval]

**Table 3:** The adjusted GEE analyses^a^ of the association between musculoskeletal pain (0–4) and work-related and individual risk factors

		All (*N* = 420)			Men (*N* = 153)			Women (*N* = 267)		
Number of pain sites		No. observations = 8820 (T0-20, 6½ years)			No. observations = 3213 (T0-20, 6½ years)			No. observations = 5607 (T0-20, 6½ years)		
		RR	95 % CI	*p*-value	RR	95 % CI	*p*-value	RR	95 % CI	*p*-value
Gender	Men	1.00								
	Women	1.20	1.02–1.42	0.02						
Mechanical workload (0–24)		1.01	1.00–1.01	<0.01	1.00	0.99–1.01	0.14	1.01	1.00–1.01	<0.01
Quantitative work demands (0–4)		1.02	0.99–1.04	0.13	0.99	0.96–1.03	0.83	1.02	1.00–1.05	0.04
Control over work intensity (0–4)		1.01	0.99–1.02	0.44	0.99	0.96–1.02	0.72	1.01	0.99–1.03	0.13
Perceived muscle tension (0–22)		1.02	1.01–1.03	<0.01	1.03	1.01–1.04	<0.01	1.02	1.01–1.03	<0.01
Handgrip strength (kg)		1.00	0.99–1.00	0.84	1.01	0.99–1.02	0.42	0.99	0.98–1.01	0.41
Muscle endurance (Seconds)		0.99	0.99–1.00	0.39	0.99	0.99–1.00	0.60	0.99	0.99–1.00	0.28
Physical activity (0–6)		0.99	0.98–1.00	0.12	0.98	0.97–1.00	0.08	1.00	0.99–1.01	0.87
Tobacco use	No	1.00			1.00			1.00		
	Yes	1.16	1.06–1.26	<0.01	1.23	1.04–1.45	0.02	1.05	0.96–1.16	0.27
Socioeconomic status	Low	1.00								
	High	0.88	0.79–0.98	0.02	0.91	0.70–1.18	0.47	0.88	0.80–0.98	0.02
Time (T0-T20)		1.01	0.99–1.01	0.05	1.01	1.00–1.01	<0.01	0.99	0.99–1.00	0.69

[RR = rate ratio, CI = 95 % confidence interval]

^a^The analyses are adjusted for the other variables listed in the table. In addition adjustments for gender were done in the analyses of all subjects

#### Gender specific

The multivariate analyses done stratified by gender showed that there were gender specific variables related to number of pain sites (Table 3). Perceived muscle tension was the only factor which was significantly associated with increased number of pain sites in both genders (men: RR = 1.03, 95 % CI 1.00–1.04, *P* < 0.01, women: RR = 1.02, 95 % CI 1.01–1.03, *P* < 0.01). For the female participants a high level of socioeconomic status was associated with a decreased number of pain sites (RR = 0.88, 95 % CI 0.88–0.98, *P* = 0.02). Mechanical workload (RR = 1.01 95 % CI 1.00–1.01, *P* < 0.01) and quantitative demands at work (RR = 1.02 95 % CI 1.00–1.05, *P* = 0.04) was risk factors for increased number of musculoskeletal pain sites among young women. For the male participants tobacco users reported 23 % increased number of pain sites compared to men not smoking or using snuff (RR = 1.23 95 % CI 1.04–1.45, *P* = 0.02). An increased leisure time physical activity was associated with a decreased number of pain sites (RR = 0.98, 95 % CI 0.97–1.00, *P* = 0.08) among young men, however not statistically significant.

## Discussion

In this prospective study we found that female gender was a risk factor for increased number of pain sites and that there was gender specific factors associated with the number of musculoskeletal pain sites among young technical school students entering working life. Perceived muscle tension was the only factor which was significantly associated with increased number of pain sites in both genders. Among young women a high level of socioeconomic status was associated with a decreased number of pain sites, while mechanical workload and quantitative demands at work was risk factors for increased number of musculoskeletal pain sites, while tobacco use was found as a risk factor for men.

During the whole study period pain from more than one body site was the most commonly reported and the number of body sites in pain was found quite stable over the 6.5 year follow-up on a group level. In our study 69 % had multisite pain at baseline, which is comparable with a group of Greek workers were the prevalence was 67 % [12]. It is also similar to the prevalence found in the general population (75 % at baseline) [13]. The pattern of reporting multiple pain sites in the general population’, has been found to be relatively stable across adulthood both in a 7 year prospective study [27] and a 14 year prospective study [13]. This is in line with our findings that the number of pain sites are rather stable. Thus, it suggests that multisite pain may persist or reoccur, meaning that those individuals who report multisite pain continues to do so. The average of pain sites appears to be settled by age 20 and little variation seem to occur thereafter [13, 28]. The findings in our young cohort of technical school students followed from they were 17–23 years of age shows similar trend. This may indicate that a pattern of pain reporting may be established early in life. Knowing that multisite pain has been found to be more severely related with work disability [6] and long term sickness absence [10], highlights the importance to identify the factors predicting the number of pain sites at an early age.

To the best of our knowledge, this is the first study to report factors associated with musculoskeletal pain at multiple body sites among students entering working life. The results however are comparable with multisite pain studies among other populations. Evidence suggest that multisite pain is more common among women than men [11, 13, 29]. This is in accordance to our results and also reflects the knowledge on gender difference in localized pain reports [30]. There have been some earlier studies concluding that mechanical workload is associated with multisite pain. A study among newly employed subjects found that new onset of widespread pain was associated with mechanical workload such as lifting, pulling, squatting and prolonged hands above shoulder height [31]. In a cross-sectional study physical load at work was also found to be strongly related to the number of painful anatomical sites reported [12]. Among Finnish industrial workers they found higher odds for multisite pain among young workers related to awkward postures and repetitive work [32]. Mechanical workload was also in our study associated with increased number of pain sites among women. Mechanical workload is also an acknowledged risk factor for localized musculoskeletal pain. However, in an earlier publication on the same data, localized neck and shoulder pain showed a weaker association compared to the association with multisite pain [14]. This may indicate that mechanical workload is a stronger predictive factor for multisite pain in comparison to localized neck and shoulder pain. The mechanical workload index used assesses a range of exposures affecting all different body regions from neck, back to arms and knees. This may help to understand why the association is more strongly related to multisite pain. Psychosocial work factors have previously been associated with multisite pain [32]. A Finnish study have found that changes in psychosocial factors especially job control over a 2 year follow-up period were associated with a higher risk of having persistent multisite pain [33]. In our study there was found an association between high quantitative job demands and number of body sites in pain, only among women. This is in contrast to other studies finding no gender difference or stronger association among men [32].

Individual factors such as smoking have been associated with increased risk of chronic pain at multiple locations in the general population [13], which is supported by the findings in our study. Perceived muscle tension was in our study found to be the most consistent risk factor for multisite pain and can be interpreted as an association between mental aspect of work as the muscle tension has been found to be influenced by the presence of psychosocial distress [34]. This is comparable to other studies that have found a higher number of pain sites among those reporting psychological distress [35]. Occupational class, educational level and wealth have all been used as indicators of socioeconomic status in earlier studies and results show socioeconomic status differences in musculoskeletal pain [13, 36]. This is in line with our findings suggesting that high level of socioeconomic status was associated with a decreased number of musculoskeletal pain sites among women.

### Methodological considerations

The main strength in this study is the longitudinal design with frequent measurements of risk factors and pain. An increasing amount of studies are now longitudinal, however considerable difference in the length of follow-up and the number of measurements are seen. Compared with other longitudinal studies our study is unique with a total of 20 follow-up measures of the outcome. The measures were done approximately every 4th month giving us the chance to assess the changes in prevalence of number of pain sites closely over time, this is beneficial as it makes the measures more robust. Knowing that musculoskeletal pain has a fluctuation nature, makes it increasingly important to have frequent follow ups. The high response rate at baseline was also a methodological strength increasing the external validity. Nevertheless, the longitudinal design led to loss to follow-up increasing the probability of selection bias. This could have resulted in an overestimation of prevalence of the number of pain sites. With background in the attrition analyses on this data [24] it can be argued that the missing were not systematical and therefore limiting selection bias. To further reduce the possible bias introduced by the missing data multiple imputation was used as it avoids the use of complete-case analysis and gives reliable and unbiased results even if the missing data are missing at random [37].

In this study both the outcome and the exposures were gathered by questionnaire, introducing a possible measurement error (except for the muscular endurance and hand grip strength). Self-reported data may be influenced by personality dimensions such as negative affectivity which can inflate the associations [38]. Spurious results can also occur if the presence of musculoskeletal pain influences the perception of work-related factors such as mechanical workload. Using a longitudinal design and analyzing the data in a way that includes adjustments for the correlation between the repeated observations within a subject was done in an attempt to minimize information bias. Multisite pain is not a well-defined term but refers to pain at more than one body site concurrently or within a defined time period. Some distinguishes between pain from the left and right side of the body [11] and some separate and include all body regions giving up to 10 pain regions [13]. Multisite pain in our study included only 4 body regions as we did not separate neck and shoulder, hip and knee or elbow and wrist. This may have led to an underestimation of the prevalence of multisite pain. However, when comparing the prevalence of number of pain sites ≥2 it was found quite similar to other populations [12, 13].

## Conclusion

The current study demonstrates that pain from multiple body sites are frequent also among young adults in their first years of working life. The knowledge on risk factors for multisite pain among young workers are sparse and this study has provided results showing that women have higher number of pain sites compared with men and that there are gender specific risk factors. Mechanical workload, quantitative demands at work and socioeconomic status was associated with number of musculoskeletal pain sites among women, while tobacco use was found as a risk factor for men. The findings from the current study increases the understanding of multisite pain among young adults. It argues that preventive measures early in working life may be of importance. The findings also reveals that the practical prevention and future research needs to take into account the gender differences in pain reports and risk factors.
